# A Rare Cause of Recurrent Pericarditis

**DOI:** 10.7759/cureus.53602

**Published:** 2024-02-05

**Authors:** Georgios Aletras, Maria Stratinaki, Maria Bachlitzanaki, Theodora Georgopoulou, Emmanouil G Foukarakis

**Affiliations:** 1 Department of Cardiology, Venizeleion General Hospital, Heraklion Crete, GRC; 2 Second Department of Internal Medicine, Venizeleion General Hospital, Heraklion Crete, GRC

**Keywords:** anakinra, rare cardiac manifestations, genetic testing, familial mediterranean fever, auto-inflammatory diseases, recurrent pericarditis

## Abstract

Recurrent pericarditis poses a significant challenge to clinicians, particularly when patients are unresponsive or intolerant to conventional treatments. Accurate diagnosis of recurrent pericarditis, potentially facilitated by collaboration with other medical specialties, is crucial for ensuring timely and appropriate treatment of symptoms and prevention of further episodes.

We present a case of a 52-year-old male patient with a history of multiple episodes of pericarditis, who was admitted to the Cardiology Department due to another recurrence. The first episode of pericarditis was diagnosed nearly a year before his current hospitalization. Initially, the patient received high doses of Ibuprofen and colchicine, but there was no favorable response to this treatment regimen. At that point, intravenous prednisolone was initiated, which led to clinical and laboratory improvement. Since then, the patient had experienced two more recurrences while tapering off prednisolone. Immunological tests, Mantoux tuberculin skin test, and chest and abdominal computed tomography (CT) had revealed no evidence of an underlying cause.

On admission the patient was febrile and the electrocardiogram showed diffuse ST elevation and PR depression in leads I, II, aVF, V2-V6. Bedside echocardiogram revealed a small pericardial effusion and since the chest X-ray was normal and no other potential infection sites were identified, the diagnosis of recurrent pericarditis was established. During his current hospitalization, intravenous prednisolone was initiated, colchicine was continued and a more detailed history was taken, raising the suspicion upon the presence of an auto-inflammatory disease. Genetic investigation identified an uncommon heterozygous mutation in the familial Mediterranean fever gene (MEFV) and after consideration of patient’s history, familial Mediterranean fever was diagnosed. Anakinra was initiated on top of colchicine and gradual tapering of corticosteroids and the patient showed significant improvement, with no other recurrence during the two-year follow-up.

## Introduction

Pericarditis refers to the inflammation of the pericardial layers and is the most common form of pericardial disease [1]. The disease can be either an isolated form or a cardiac manifestation of a systemic disorder. According to current European Society of Cardiology (ESC) guidelines, the diagnosis of acute pericarditis requires at least two of the following criteria: pleuritic chest pain, pericardial rub, new widespread ST elevation or PR depression on ECG, and new or worsening pericardial effusion. Elevation of inflammatory markers, i.e. C-reactive protein (CRP), erythrocyte sedimentation rate (ESR) and white blood cell count elevation (WBC) and evidence of pericardial inflammation by an imaging technique (computed tomography scan, or cardiac magnetic resonance) may help the diagnosis and the monitoring of disease activity [1,2].

Recurrent pericarditis is the most common complication of acute pericarditis and its diagnosis is established according to the same criteria as the first episode and a symptom-free interval of four to six weeks or longer [2,3]. First recurrence occurs in 15-30% of cases of acute idiopathic pericarditis. Of these patients, a second recurrence occurs in 25-50% of cases, a third in 20-40% and multiple recurrences in 5-10%, with the last subgroup being the most challenging in its management [4].

The etiology of recurrent pericarditis is believed to be immune-mediated, linked to incomplete treatment rather than recurrent viral infections. Evidence supporting this includes the timing of recurrences, the presence of non-organ-specific antibodies and positive response to corticosteroid therapy. Despite efforts to identify specific causes, many cases are labeled "idiopathic", reflecting the inability to establish a precise etiology. However, determining the underlying cause, especially in patients with multiple recurrences, can lead to improved management and definitive treatment [1,5].

Regarding medical treatment, Aspirin (750mg-1g every eight hours for one to two weeks) or non-steroidal anti-inflammatory drugs (NSAIDs) at a dose of 600-800mg every eight hours for one to two weeks are typically the first-line therapy for recurrent pericarditis. Colchicine (0.5-1.2mg in one or divided doses for at least six months) is recommended in addition to standard anti-inflammatory therapy, using weight-adjusted doses without a loading dose. In cases where there is an incomplete response to aspirin/NSAIDs and colchicine, corticosteroids (prednisone at a dose of 0.2-0.5mg/kg/day) may be added as a second-line treatment option. The use of corticosteroids should be limited to patients with specific indications, such as systemic inflammatory diseases, pregnancy, NSAIDs treatment failure or contraindications (true allergy, recent peptic ulcer or gastrointestinal bleeding, or oral anticoagulant therapy with high bleeding risk) as they are associated with higher recurrences rates. Corticosteroid tapering should proceed gradually, with dose reductions made only in asymptomatic patients exhibiting very low CRP levels. For patients requiring high long-term doses of corticosteroids or those who do not respond to anti-inflammatory therapies, alternative drugs such as intravenous immunoglobulin (IVIG) and interleukin-1 (IL-1) receptor antagonists have been used, although strong evidence-based data are lacking [1].

This paper presents a clinical case of recurrent pericarditis in a patient with frequent hospitalizations, where a collaborative effort involving Rheumatologists led to the diagnosis of familial Mediterranean fever (FMF) as the underlying cause. FMF is an autosomal recessive autoinflammatory disorder primarily affecting populations from the Mediterranean basin. It is characterized by recurrent fever episodes accompanied by inflammation of serosal membranes, including peritonitis, pleuritis, arthritis, and pericarditis. Although rare, FMF can manifest with cardiac involvement, mainly related to systemic inflammation or secondary amyloidosis [6]. The minimal and most current criteria for diagnosis of FMF are the Tel-Hashomer clinical criteria [7,8].

The case highlights the importance of considering auto-inflammatory diseases as a potential etiology in recurrent pericarditis cases, especially in patients with early onset of the disease, failure of colchicine therapy and need for immunosuppression to control the disease [4]. For these challenging cases, the interdisciplinary collaboration between Cardiologists and other subspecialties such as Rheumatologists can identify underlying causes and optimize patient management.

## Case presentation

A 52-year-old Caucasian male visited the Emergency Department (ED) due to low-grade fever and pleuritic chest pain for five days. The patient had a history of liver haemangioma, gastroesophageal reflux disease (GERD) due to esophageal achalasia and recurrent pericarditis and he was on tapering with a very low dose of prednisolone (5mg/d) and colchicine (1mg/day). There was no family history of pericarditis or any autoimmune/auto-inflammatory disease in first- and second-degree relatives. Additionally, the patient had never complained of arthralgias/myalgias or skin rash.

The patient had been first hospitalized nearly a year ago for acute pericarditis treatment. Initial treatment with Ibuprofen (2400mg/day) and colchicine (1mg/day) yielded no satisfactory response, prompting the initiation of prednisolone during that time. Considering the patient's weight of 80kg, 40mg of intravenous prednisolone was administered in addition to colchicine. Subsequent management with low-dose corticosteroids and oral colchicine led to an improvement in his condition. Following a nine-day hospital stay, he was discharged with complete resolution of symptoms.

Seven months post discharge, the patient was brought to the ED complaining of chest pain and flu-like symptoms with low-grade fever and excessive fatigue. The laboratory tests showed raised inflammation markers, while the transthoracic echocardiogram showed small pericardial effusion around the lateral wall of the left ventricle, with no signs of constriction. Based on his clinical picture, recurrent pericarditis was diagnosed and the patient was admitted to the Cardiology Department. At that time, immunological control including antinuclear antibodies (ANA), antineutrophil cytoplasmic antibodies (ANCA), extractable nuclear antigen (ENA) test, rheumatoid factor, complement fractions (C3, C4) and tumor marker tests, Mantoux tuberculin skin test and chest and abdominal computed tomography (CT) were performed, which showed no evidence of an underlying cause. After a few days on intravenous prednisolone (40mg/day), the patient showed significant clinical and laboratory improvement and he was discharged with instructions for a slower tapering of prednisolone and continuation of colchicine (1mg/day).

Four months after the treatment of recurrent pericarditis, the patient returned to the ED with the same symptoms as the previous times and he was hospitalized once again. Intravenous prednisolone (40mg/day) was administered and the patient was discharged six days after admission, while he was advised to visit a Rheumatologist.

On his current examination, the patient was febrile (T=38^o^C), hemodynamically stable (blood pressure: 134/72mmHg, 60 beats per minute), with good oxygen saturation on room air. The lung examination did not reveal any abnormalities, while pericardial friction rub was present on auscultation. The ear, nose and throat as well as neurological exam was normal.

**Figure 1:**
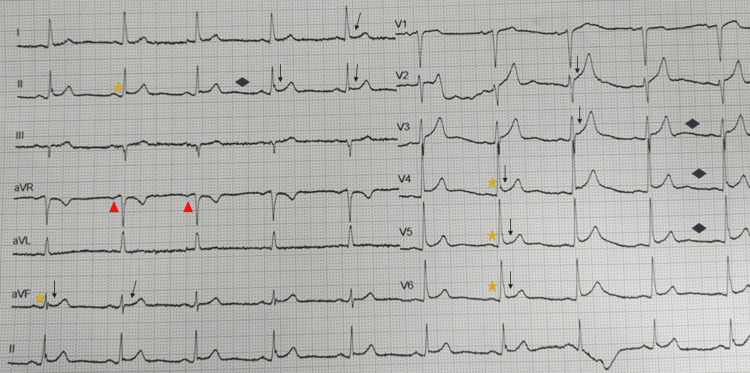
ECG demonstrating sinus rhythm with widespread concave ST elevation (black arrows) and PR depression (yellow stars) throughout most limb leads (I, II, aVF) and precordial leads (V2-6), but also PR elevation in lead aVR (red triangles) and Spodick’s sign (blue diamonds)

The ECG demonstrated sinus rhythm with widespread concave ST elevation and PR depression throughout most of the limb leads (I, II, aVF) and precordial leads (V2-6), but also PR elevation in lead aVR and Spodick’s sign, which is the downsloping of the TP segment (Figure 1). The arterial blood gases (ABGs) revealed insignificant respiratory alkalosis (ABGs-fraction of inspired oxygen (FiO2) 21%: pH 7.47, partial pressure of carbon dioxide (pCO2) 34.7mmHg, partial pressure of oxygen (pO2) 82mmHg, bicarbonate (HCO3) 24.9mEq/L, lactate 0.3mmol/L) and the laboratory tests showed markedly raised inflammation markers (WBC 12.500/μL, normal values: 4.500-10.000/μL, CRP 27.22pg/ml, normal values <0.5pg/ml). High-sensitive cardiac troponin, renal function and urinalysis were within the normal limits.

**Figure 2:**
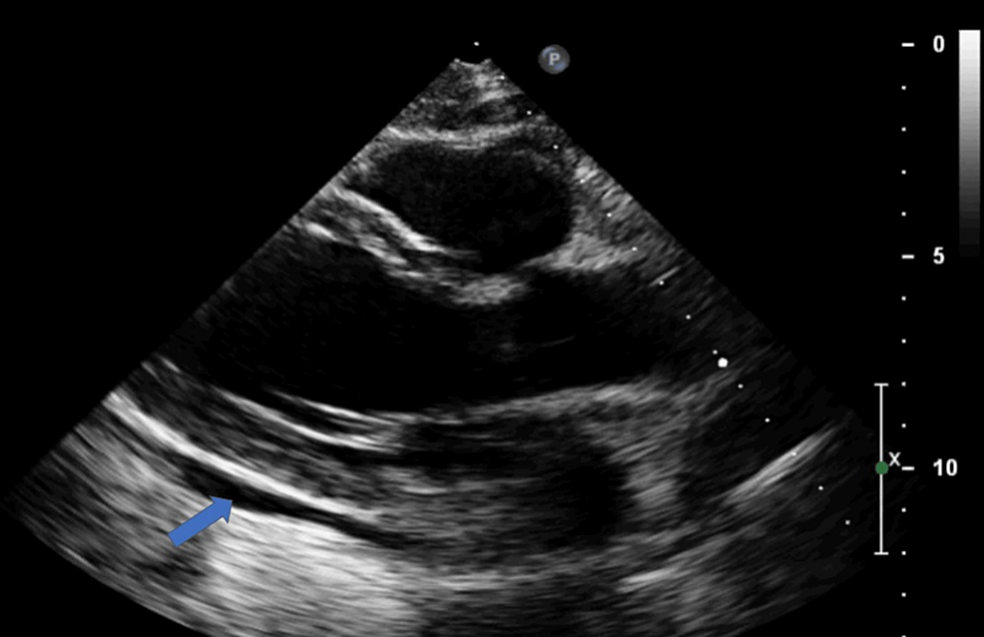
Transthoracic echocardiogram showing small pericardial effusion around the lateral wall of the left ventricle (blue arrow).

**Figure 3:**
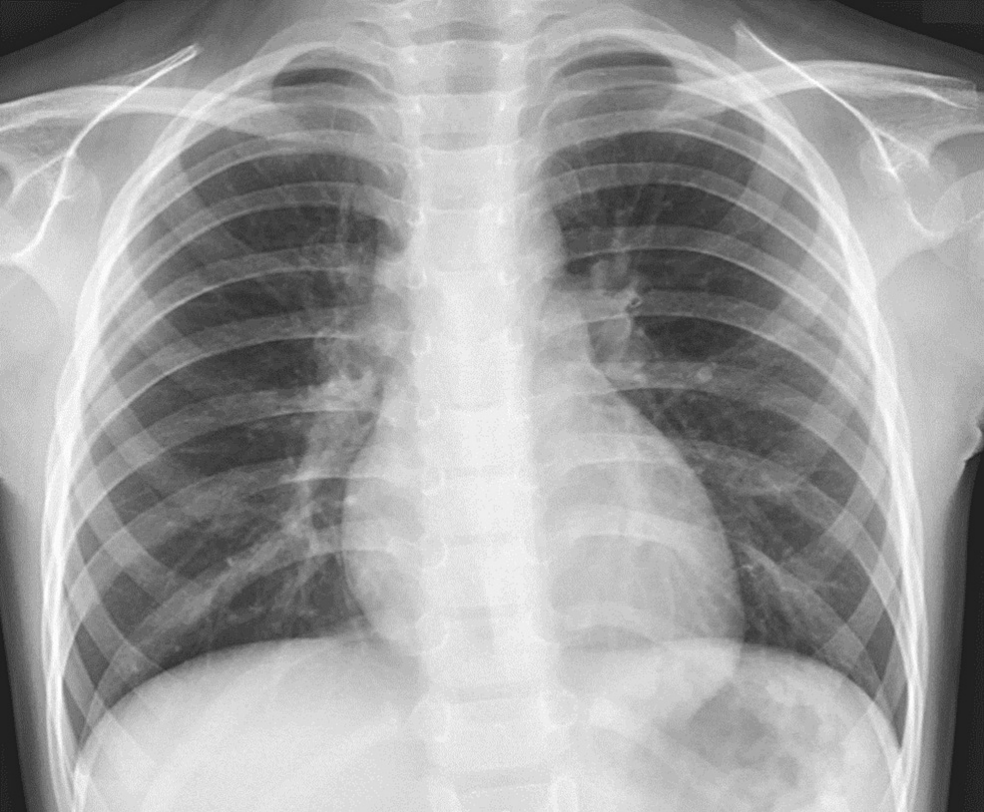
Chest X-ray showing no signs of respiratory tract infection.

Blood and urine cultures were taken and a bedside echocardiogram was performed which showed small pericardial effusion around the lateral wall of the left ventricle with normal size and function of the left and right ventricle (Figure 2), no regional abnormalities and absence of signs of constriction and tamponade. Rapid and polymerase chain reaction coronavirus disease 2019 (COVID-19) tests were negative and the chest X-ray did not reveal any signs of infection (Figure 3). The patient was admitted to the Cardiology Department for further management.

Based on the 2015 ESC Guidelines for the diagnosis and management of pericardial diseases, the diagnosis of recurrent pericarditis was made. Blood and urine cultures were negative and as the patient had a good response only to corticosteroids in his previous hospitalizations, low-dose of intravenous prednisolone (40mg/day) and oral colchicine (1mg/day) were initiated. During his hospital stay, a more thorough history was taken and the patient mentioned recurrent episodes of self-limiting fever during his childhood, while at the age of 12, he was hospitalized with sudden onset of fever and severe abdominal pain, which was attributed to a gastrointestinal tract infection. Based on the above, suspicion of auto-inflammatory diseases was raised. The most common auto-inflammatory diseases responsible for recurrent pericarditis are FMF and tumor necrosis factor receptor-associated periodic syndrome (TRAPS). At that time a Rheumatologist was advised and molecular genetic testing was performed, including MEFV and tumor necrosis factor receptor superfamily member 1A (TNFRSF1A) genes.

**Figure 4:**
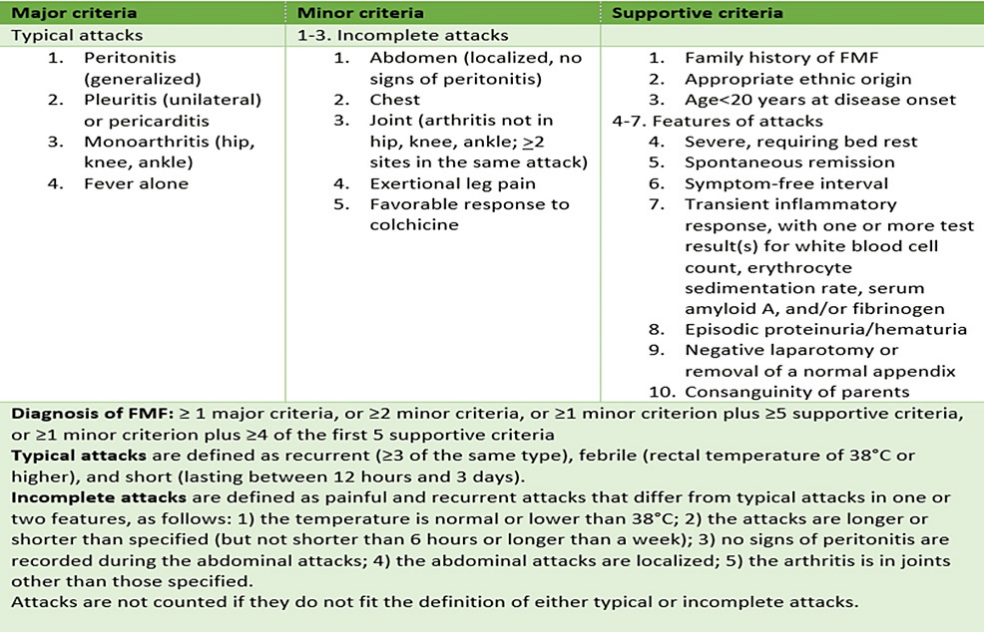
Tel-Hashomer criteria for the diagnosis of familial Mediterranean fever [8]

Molecular genetic testing identified a heterozygous mutation for M680I in the MEFV gene and considering the patient’s clinical picture and the Tel-Hashomer criteria (Figure 4), the diagnosis of FMF was reached. Anakinra, an IL-1 inhibitor, was initiated and the patient exhibited significant improvement in his clinical and laboratory parameters. The patient was discharged from the Cardiology Department without any symptoms a few days later and received instructions for a gradual tapering of corticosteroids, continuation of colchicine and anakinra, while a re-evaluation visit at Rheumatology and Cardiology outpatient office was scheduled. The patient remained under close medical observation and two years later remains asymptomatic, without a new episode of recurrent pericarditis.

## Discussion

Recurrent pericarditis is a significant clinical challenge due to its propensity for recurrence and potential complications, such as compressive pericarditis and cardiac tamponade. The frequency of complications varies depending on the underlying cause, while its presence has a significant impact on the quality of life and psychological well-being, due to the symptomatic episodes and the need for constant medical assistance. The presented case underscores the importance of a thorough diagnostic evaluation in recurrent pericarditis cases, as well as the role of interdisciplinary collaboration in identifying underlying causes and optimizing patient management.

In this case, thorough history-taking and molecular genetic testing played a pivotal role in confirming the diagnosis of FMF. The detailed patient history, including recurrent episodes of self-limiting fever during childhood, recurrences of pericarditis and hospitalization at the age of 12 due to sudden onset of fever and severe abdominal pain provided crucial clues suggestive of an underlying inflammatory disorder. This underscores the critical role of thorough history-taking in recognizing atypical presentations and guiding diagnostic evaluation, ultimately leading to the consideration of molecular genetic testing to confirm the diagnosis of FMF.

The occurrence of pericarditis as a manifestation of FMF is a topic of debate. Sohar et al. reported no cases of pericarditis in their study of 470 FMF patients [9,10], but several other studies have found a higher incidence of pericarditis among FMF patients compared to the general population. For instance, Kees et al. observed a prevalence of 0.7% in a retrospective study of 4000 FMF patients over 20 years [11], while Dabestani et al. reported a much higher prevalence of pericarditis (27%) in FMF patients [12]. Pericardial involvement in FMF typically occurs later in the disease course, typically presenting as chest pain lasting around four days and resolving spontaneously. Severe cases with massive pericardial effusions and cardiac tamponade requiring pericardiocentesis have also been documented in the literature [10]. In our case, acute recurrent pericarditis was the only manifestation of FMF, a scenario rarely documented in literature.

While FMF is considered an autosomal recessive disease, recent studies suggest the existence of a significant subset of patients with FMF who harbor only one MEFV mutation. In such cases, the detection of a single mutation appears to be adequate in the presence of clinical symptoms for diagnosing FMF, as observed in our patient's case [7].

The identification of a heterozygous mutation for M680I in the MEFV gene provided definitive evidence supporting the diagnosis of FMF, highlighting the value of collaboration with other subspecialties and genetic testing in establishing a precise diagnosis and guiding targeted treatment strategies in patients with recurrent pericarditis.

The management of recurrent pericarditis often involves a combination of pharmacological therapies aimed at reducing inflammation and preventing recurrences. In this case, the initiation of anakinra, an IL-1 inhibitor, resulted in a significant improvement in clinical and laboratory results and led to sustained remission of symptoms and absence of recurrent pericarditis episodes during a two-year follow-up period. This highlights the importance of early diagnosis and targeted treatment in improving long-term outcomes and preventing disease recurrence in patients with FMF-related or other auto-inflammatory disease-related recurrent pericarditis.

## Conclusions

This case highlights the complexity of recurrent pericarditis and the importance of comprehensive history-taking, interdisciplinary collaboration, and targeted treatment approaches in optimizing patient management. Further research is warranted to elucidate the underlying mechanisms and identify novel therapeutic targets, especially in the context of cardiac manifestations related to auto-inflammatory diseases.
